# Deterioration to extinction of wastewater bacteria by non-thermal atmospheric pressure air plasma as assessed by 16S rDNA-DGGE fingerprinting

**DOI:** 10.3389/fmicb.2015.01098

**Published:** 2015-10-06

**Authors:** Wael S. El-Sayed, Salama A. Ouf, Abdel-Aleam H. Mohamed

**Affiliations:** ^1^Biology Department, Faculty of Science, Taibah UniversityAlmadinah Almunawarah, Saudi Arabia; ^2^Microbiology Department, Faculty of Science, Ain Shams UniversityCairo, Egypt; ^3^Botany and Microbiology Department, Faculty of Science, Cairo UniversityGiza, Egypt; ^4^Physics Department, Faculty of Science, Taibah UniversityAlmadinah Almunawarah, Saudi Arabia; ^5^Physics Department, Faculty of Science, Beni-Suef UniversityBeni Suef, Egypt

**Keywords:** dielectric barrier discharge plasma, wastewater, DGGE, 16S rDNA, bacterial community

## Abstract

The use of cold plasma jets for inactivation of a variety of microorganisms has recently been evaluated via culture-based methods. Accordingly, elucidation of the role of cold plasma in decontamination would be inaccurate because most microbial populations within a system remain unexplored owing to the high amount of yet uncultured bacteria. The impact of cold atmospheric plasma on the bacterial community structure of wastewater from two different industries was investigated by metagenomic-based polymerase chain reaction-denaturing gradient gel electrophoresis (DGGE) utilizing 16S rRNA genes. Three doses of atmospheric pressure dielectric barrier discharge plasma were applied to wastewater samples on different time scales. DGGE revealed that the bacterial community gradually changed and overall abundance decreased to extinction upon plasma treatment. The bacterial community in food processing wastewater contained 11 key operational taxonomic units that remained almost completely unchanged when exposed to plasma irradiation at 75.5 mA for 30 or 60 s. However, when exposure time was extended to 90 s, only *Escherichia coli*, Coliforms, *Aeromonas* sp., *Vibrio* sp., and *Pseudomonas putida* survived. Only *E. coli, Aeromonas* sp., *Vibrio* sp., and *P. putida* survived treatment at 81.94 mA for 90 s. Conversely, all bacterial groups were completely eliminated by treatment at 85.34 mA for either 60 or 90 s. Dominant bacterial groups in leather processing wastewater also changed greatly upon exposure to plasma at 75.5 mA for 30 or 60 s, with *Enterobacter aerogenes, Klebsiella* sp., *Pseudomonas stutzeri*, and *Acidithiobacillus ferrooxidans* being sensitive to and eliminated from the community. At 90 s of exposure, all groups were affected except for *Pseudomonas* sp. and *Citrobacter freundii*. The same trend was observed for treatment at 81.94 mA. The variability in bacterial community response to different plasma treatment protocols revealed that plasma had a selective impact on bacterial community structure at lower doses and potential bactericidal effects at higher doses.

## Introduction

Industrial, agricultural, and domestic wastewater must be treated to eliminate pathogenic microorganisms and prevent their transmission through the environment. However, conventional wastewater treatment processes do not guarantee disinfection and elimination of these organisms (Howard et al., 2004). Moreover, discharging inefficiently treated wastewater to the environment results in environmental and health problems such as eutrophication, oxygen consumption, and toxicity (Ding et al., 2011). Accordingly, there is a continuous demand for alternative highly efficient methods of treatment to enable complete elimination of bacteria from wastewater.

Non-thermal atmospheric pressure plasmas (APPs) have been recognized as a new paradigm in biomedical applications and materials processing. APP systems are considered cost-effective and convenient alternatives to low-pressure plasma systems. APPs are vacuum-less generated plasmas with gas temperatures much lower than the electron temperature, even approaching room temperature. Owing to their low temperature and absence of a vacuum, APPs have widespread physical, chemical, and biomedical applications. In particular, low temperature APPs (Becker et al., 2005) have the potential for application in decomposition or detoxification of gaseous materials, surface treatment, sterilization, protein destruction, decontamination, food processing, teeth bleaching, dental cavity treatment, blood coagulants, treatment of living tissue, wound care, deposition, etching, and synthesis of carbon nano-tubes, sources of UV and excimer radiation, and as reflectors or absorbers of electromagnetic radiation (Deng et al., 2007a,b; Harbec et al., 2007; Fridman et al., 2008; Niemira and Sites, 2008; Daeschlein et al., 2009; Kong et al., 2009; Lee et al., 2009; Lloyd et al., 2009; Choi et al., 2010; Yasuda et al., 2010).

Non-thermal plasma is partially ionized gas with ions, electrons, and uncharged particles such as atoms, molecules, and radicals that have a variety of applications. Atmospheric pressure non-thermal plasma (cold plasma) sterilization is a promising technique that could be regarded as an alternative to other conventional sterilization methods such as high temperature sterilization, ethylene oxide sterilization and sterilization by radiation. The major components of plasmas are reactive species (OH, NO, O), charged particles, and UV photons; therefore, they have been employed in sterilization of a wide range of Gram-positive and Gram-negative bacteria, viruses, and fungi (Leclaire et al., 2008; Ermolaeva et al., 2012). Cold atmospheric plasma (CAP) is a specific type of plasma that is less than 104°F at the point of application. The discharge of CAP results in generation of a wide range of reactive species responsible for antimicrobial effects; accordingly, it has been effective for microbial inactivation (Deng et al., 2007a,b; Zhang et al., 2013; Ziuzina et al., 2013, 2014). Currently available reports have concentrated on the effects of CAP on microbial activities using clinical samples and discussed possible uses of CAP as a sterilizing agent. However, there is no available literature describing the impact of CAP on microbial community structures in ecological systems.

Traditional culture-based approaches to analysis of bacterial communities and diversity have recently been regarded as being inappropriate because of strong evidence that this approach detects only a small proportion (less than 1%) of the bacteria present owing to the selectivity of growth media and conditions (Ward et al., 1990). Owing to the existence of yet uncultured microorganisms in a system, the actual effects of plasma treatment on such systems remain uncertain. Analyses of bacterial community structure have recently been performed using metagenomic molecular-based approaches (Amann et al., 1995; Head et al., 1998; Hugenholtz et al., 1998). As an alternative to culture-based methods, molecular-based methods such as 16S rDNA clone libraries (Otawa et al., 2006), restriction fragment length polymorphism (Gilbride et al., 2006b), repetitive extragenic palindromic polymerase chain reaction (PCR) (Baker et al., 2003) and fluorescence *in situ* hybridization (Bjørnsson et al., 2002) have been applied to investigation of wastewater associated microbial communities. PCR-DGGE (denaturing gradient gel electrophoresis; Muyzer et al., 1993) has been regarded as a particularly powerful genetic fingerprinting technique for evaluation of bacterial community structures in different environmental niches, and has been used successfully to describe bacterial communities associated with some wastewater systems (Boon et al., 2002; Casserly and Erijman, 2003; Kaksonen et al., 2003; Gilbride et al., 2006a,b).

This study was conducted to apply PCR-DGGE to investigate the impact of atmospheric pressure dielectric barrier discharge (DBD) cold plasma on wastewater bacterial community structure (metagenome) and dynamics for possible application in wastewater treatment facilities.

## Materials and Methods

### DBD Plasma System

**FIGURE 1:**
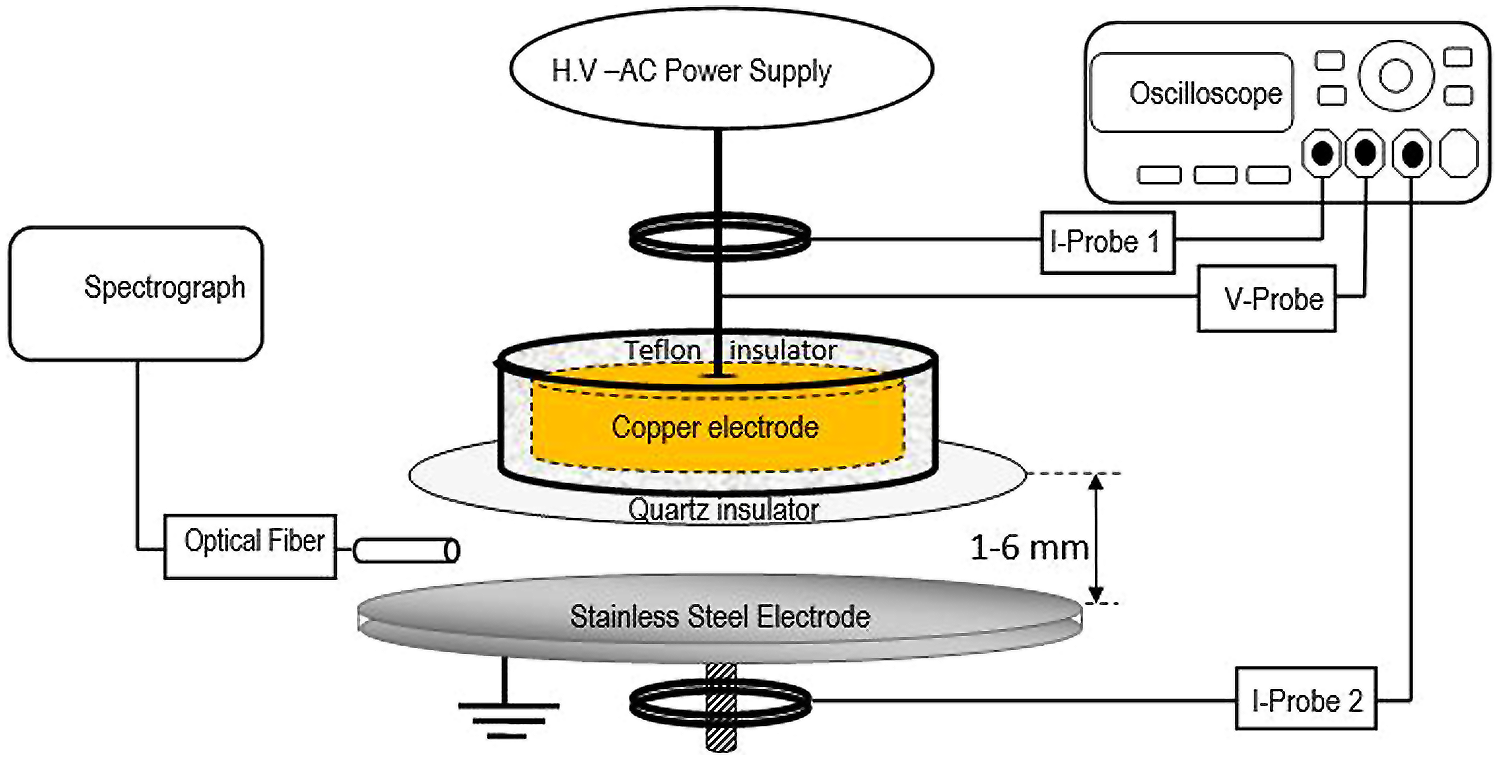
**Schematic diagram of experimental set up of atmospheric pressure dielectric barrier discharge (DBD) plasma in air**.

The non-thermal atmospheric pressure DBD air plasma system consists of two parallel metallic electrodes separated by a 4-mm gap (**Figure 1**). The upper electrode has a diameter of 45 mm, is made of copper and is covered from the bottom by a 2-mm-thick 80 mm × 80 mm dielectric alumina sheet. This electrode is connected to a high voltage power-source that can supply a sinusoidal waveform signal with a maximum 30 kV and 40 kHz output. The other sides of the powered electrode are covered by insulator (Teflon) to protect the users. The lower electrode is a stainless steel disk with a diameter of 45 mm that is grounded. The voltage and current waveform are monitored using a DPO7354 C-3.5 GHz Tektronix oscilloscope with a P6015A-1:1000 Tektronix-high voltage probe and a personal probe (model: 6585), respectively. Two current probes were used, I-probe 1 to monitor the current through the high voltage electrode (total current) and I-probe 2 to monitor the current through the ground electrode (ground current). Lissajous figure, the charge–voltage (Q–V) characteristics were estimated using a capacitance C means equal 15 nF as explained previously (Eliasson and Kogelschatz, 1991; Kogelschatz et al., 1997; Wagner et al., 2003). The samples were placed on the lower stainless steel grounded electrode for treatment and a Nikon digital camera D3200 with an AF-S Micro NIKKOR 105 mm lens was used to capture the visible plasma images. Plasma emission spectra were investigated using a 0.5 m imaging triple grating SP2500i monochromator/spectrograph coupled with a 3 m fiber optics bundle. The spectrograph has three gratings, 3600, 1800, and 150 G/mm, which are blazed at 240, 500, and 500 nm, respectively. The spectrograph has a built-in high sensitive photomultiplier detector (model ARC-P2, Princeton instrument) with a sensitivity range of 190–900 nm. The fiber is placed at the middle distance between the electrodes 3 mm from the plasma edge.

### Wastewater Samples, Plasma Treatment, and Viable Cell Counts

Wastewater samples were collected from a food processing and a leather processing plant in sterile bottles and transferred immediately to laboratory for plasma treatment. Aliquots of water samples were placed on sterilized stainless steel electrodes and subjected to different doses of air DBD plasma treatments. The plasma treatment dosages were adjusted to three different peak to peak average discharge current conditions (75.5, 81.94, and 85.34 mA), and there were three different exposure times (30, 60, and 90 s). Viable cell counts were taken before and after plasma treatments. Control and treated samples were serially diluted in saline Phosphate Buffer (PBS), 100 μl of each dilution were spread in triplicate onto Luria-Bertani (LB) agar plates. The numbers of colony-forming units (cfu/ml) were determined after 24 h of incubation at 37°C. Another portion of plasma treated wastewater samples were kept in sterile polypropylene tubes at 4°C for 24 h before DNA extraction.

### DNA Extraction and PCR Amplification of 16S rRNA Genes

Metagenomic DNA was extracted using an Ultra Clean Soil DNA purification kit (Mo Bio Laboratories, Solana Beach, CA, USA). Treated samples were filtered through 0.22 μm Millipore bacterial filters. The whole filter film with retained bacteria was transferred to bead beating tubes and vortexed horizontally for 1 min at room temperature. The supernatant was then collected and DNA was precipitated and purified according to the manufacturer’s instructions. Amplification of 16S rRNA genes for DGGE analysis was performed using GC-clamp primers (EUB341F-GC: 5′-CGCCCGCCGCGCGCGGCGGGCGGGGCGGGGGCACGGGGGGCCTACGGGAGGCAGCAGCAG-3′ and EUB517R: 5′-ATTACCGCGGCTGCTGG-3′) that corresponded to positions 341 and 517 in *Escherichia coli* (Muyzer et al., 1993). Amplification was performed in 25 μl reaction mixtures composed of 2.5 μl of 10x *Taq* buffer (100 mM Tris-HCl, pH 8), 1.25 mM MgCl_2_, 100 μM dNTPs (Invitrogen, USA), 1.2 μM forward and reverse primer (Invitrogen, USA), 0.5 U *Taq* DNA polymerase (Invitrogen, USA), and about 5 ng of template DNA. PCR was performed in an Applied Biosystem Thermal Cycler (Model 2720, USA). A touchdown PCR program was implemented as follows: initial denaturation at 95°C for 5 min, followed by five cycles of 94°C for 40 s, annealing at 65°C for 40 s, and extension at 72°C for 40 s; five cycles of 94°C for 40 s, annealing at 60°C for 40 s, and extension at 72°C for 40 s; 10 cycles of 94°C for 40 s, annealing at 55°C for 40 s, and extension at 72°C for 40 s; 10 cycles of 94°C for 40 s, annealing at 50°C for 40 s, and extension at 72°C for 40 s and then a final hold at 72°C for 7 min. Amplicons were analyzed by electrophoresis on 1% agarose gels with size markers (1 kb DNA ladder, Invitrogen, USA), then visualized using ethidium bromide.

### Denaturing Gradient Gel Electrophoresis

Denaturing gradient gel electrophoresis was performed using a Dcode Mutation Detection System (BioRad Laboratories Ltd., Hertfordshire, UK). PCR products were electrophoresed with 0.5x TAE buffer (1x TAE buffer is 0.04 M Tris base, 0.02 M sodium acetate, and 10 mM EDTA [pH 7.4]) on 8% acrylamide gel containing a 25 to 50% denaturating gradient of formamide and urea. DGGE was conducted at 60°C for 5 h at 200 V. The gel was then treated with SYBR Green I Nucleic acid gel stain (Cambrex Bio Science Rockland, USA), photographed and analyzed for DGGE band profile using an UV gel documentation system (BioRad Laboratories Inc., Hercules, CA, USA).

### Sequencing of DGGE Bands

Dominant DGGE bands were cut off with a sterile scalpel and eluted by incubation in 100 μl of TE buffer at 100°C for 5 min. The supernatant was then used as a template for PCR amplification. Reamplification of 16Sr RNA genes from excised DNA fragments was performed using bacterial primers EUB314F without the GC clamp and EUB517R. Amplification was verified by electrophoresis on 1% agarose gel. PCR products were directly sequenced using a BigDye terminator cycle sequencing method (Sanger et al., 1977) at the GenoScreen sequencing facility (Genoscreen, Lille, France).

### Numerical Analysis of DGGE Fingerprints

The DGGE fingerprints were analyzed using a Quantity One 1D software (BioRad). The total number of DGGE bands was used to represent OTUs richness (Duarte et al., 2009). Bacterial diversity was estimated based on densitometric measurements and Shannon diversity index (*H*′) (Duarte et al., 2009; Ping et al., 2010), Equation (1):

$$\begin{array}{c} H^{\prime }=-\Sigma P_{i}(\ln P_{i})\\ P_{i}=n_{i}/N_{i}\; \end{array}$$

*P_i_* is a relative intensity of DNA band in the fingerprint, *n_i_* is densitometrically measured intensity of individual DNA band, and *N_i_* is the total amount of DNA in the fingerprint. The relative intensity of each band (*P_i_*) was used to express the relative frequency of each phylotype (Moreirinha et al., 2011).

### Sequence Analysis

The sequences obtained from the 16S rRNA genes were analyzed using *Genetyx-Win* MFC application software version 4.0. The reference 16S rRNA gene sequences were retrieved from the GenBank database (National Center for Biotechnology Information, National Library of Medicine, USA). Sequences were compared with their closest matches in GenBank by nucleotide-nucleotide BLAST searches to obtain the nearest phylogenetic neighbors (www.ncbi.nlm.nih.gov/BLAST/).

### Nucleotide Sequence Accession Number

The 16S rDNA sequences identified in this study have been deposited in the GenBank database under accession numbers LC011117–LC011137.

## Results

### DBD Plasma Characterization

**FIGURE 2:**
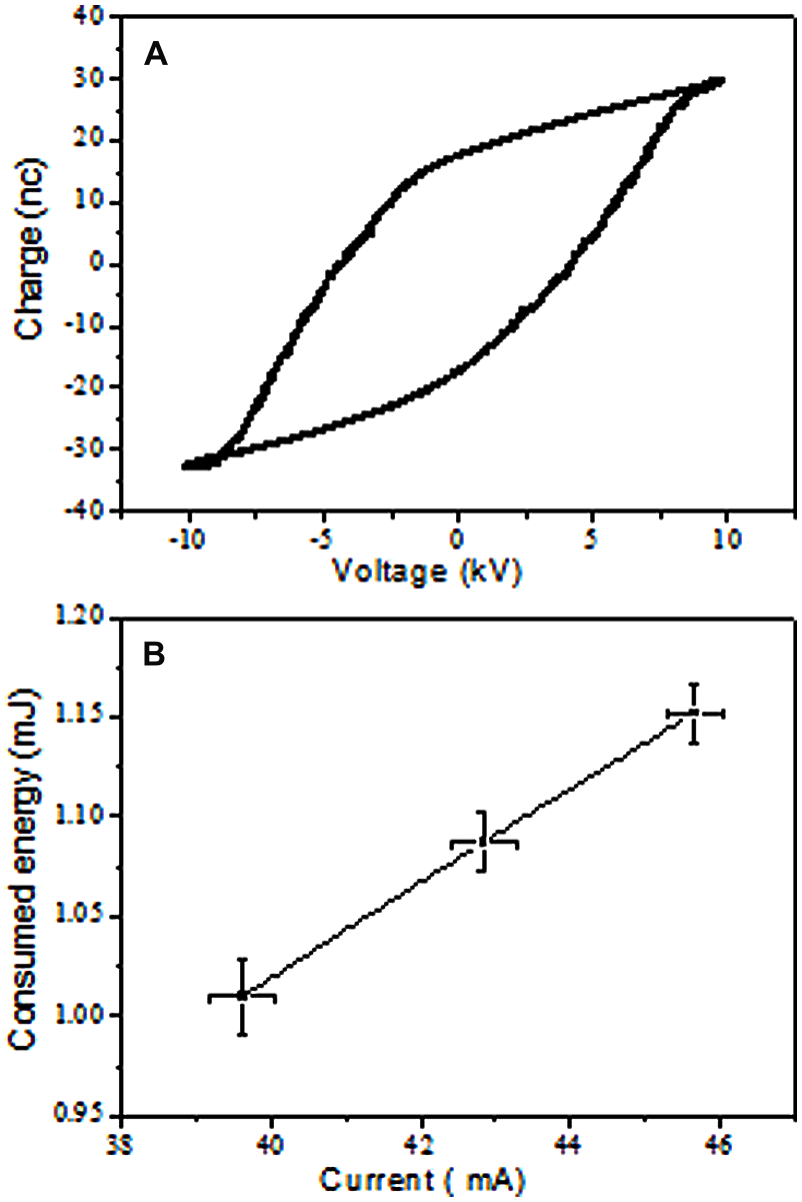
**Measurement of DBD consumed energy. (A)** Example of charge – Voltage Lissajous Figure, the calculated consumed energy was 1.020 mJ at 20.24 KV and 40.35 mA peak to peak voltage and total current using 15 nF capacitance. **(B)** Increase in consumed energy with total current.

The voltage and current waveforms presented in the Supplementary Figure S1, illustrated that the generated plasma was inhomogeneous and contained micro-discharges or streamers. Once the discharge breakdowns started (∼12 kV), flow occurred from many points via the development of micro-discharges as illustrated by the total and ground currents. The plasma started to fill the 4-mm gap between the two electrodes as the applied voltage increased, which is typical for DBD plasma (Kogelschatz et al., 1997). The plasma homogeneity increased visually with decreasing gap distance or increasing applied voltage due to the diffusivity and interference from the micro-discharges. **Figure 2A** shows a Lissajous figure of the charge-voltage characteristics, which were used to estimate the energy consumed by DBD plasma by measuring its enclosed area. The consumed energy was investigated based on the average discharge peak to peak current (**Figure 2B**). The consumed power can ultimately be calculated by multiplying energy consumed by the discharge frequency (25 kHz). The atmospheric pressure air DBD plasma consumed energy increased with increasing discharge current. This increase in consumed energy was because of increasing loss of charge carriers in response to enlargement of the DBD plasma volume with increasing discharge current. Once the DBD covered the electrodes completely and filled the discharge gap, the discharge current density increased with further increases in discharge current. This increase in current density increased the electron density, leading to increasing loss of the charge carrier (diffusion and recombination). These losses in the discharge carrier were compensated for by increases in the discharge consumed energy.

**FIGURE 3:**
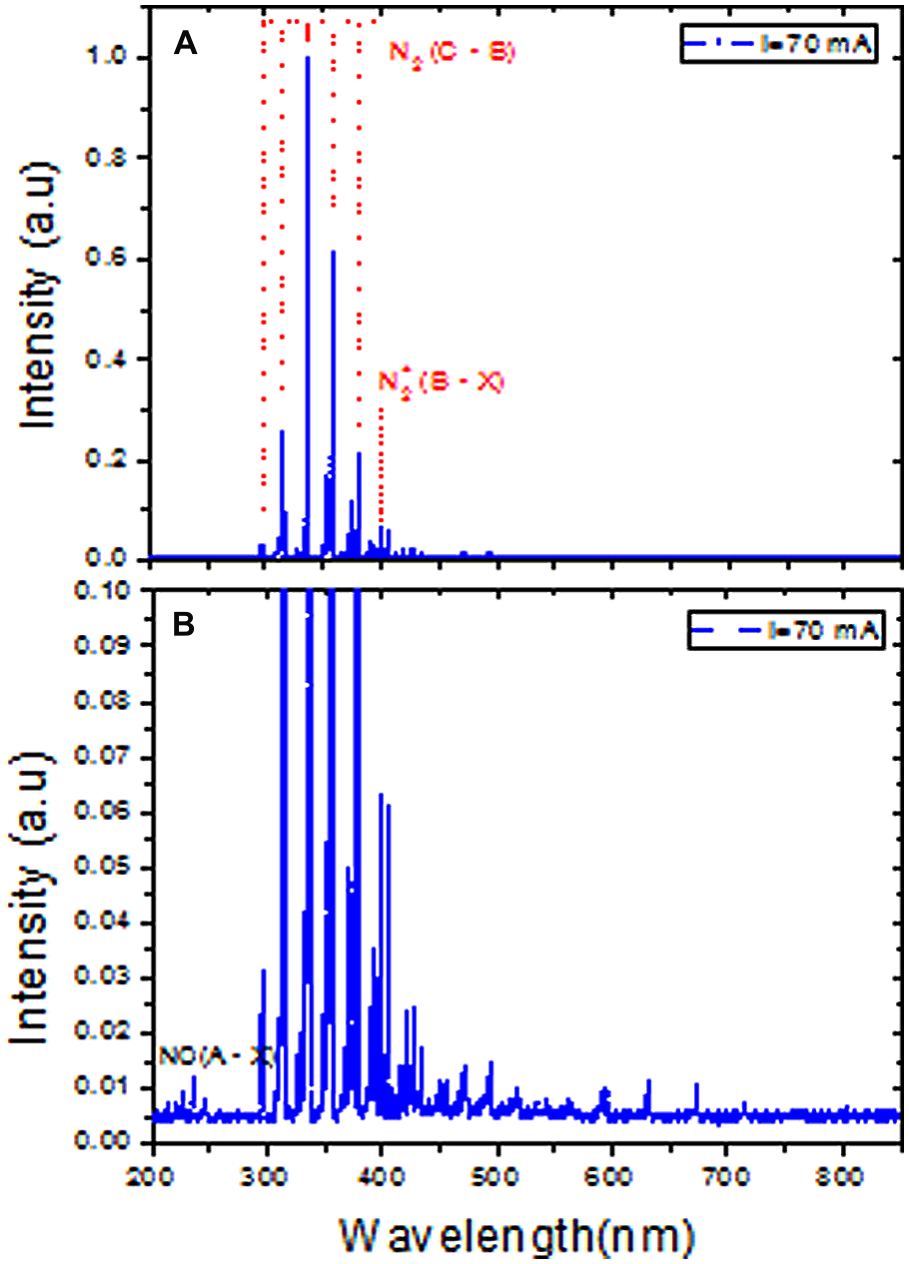
**Emission spectra of atmospheric pressure DBD plasma at 70 mA peak to peak current in the range of 200 to 850 nm. (A)** Spectra were normalized to the highest peak of the N_2_ second positive system 0–0 transition (337.2 nm). **(B)** Represents magnified scale to show the existence of nitric oxide (NO) species.

**FIGURE 4:**
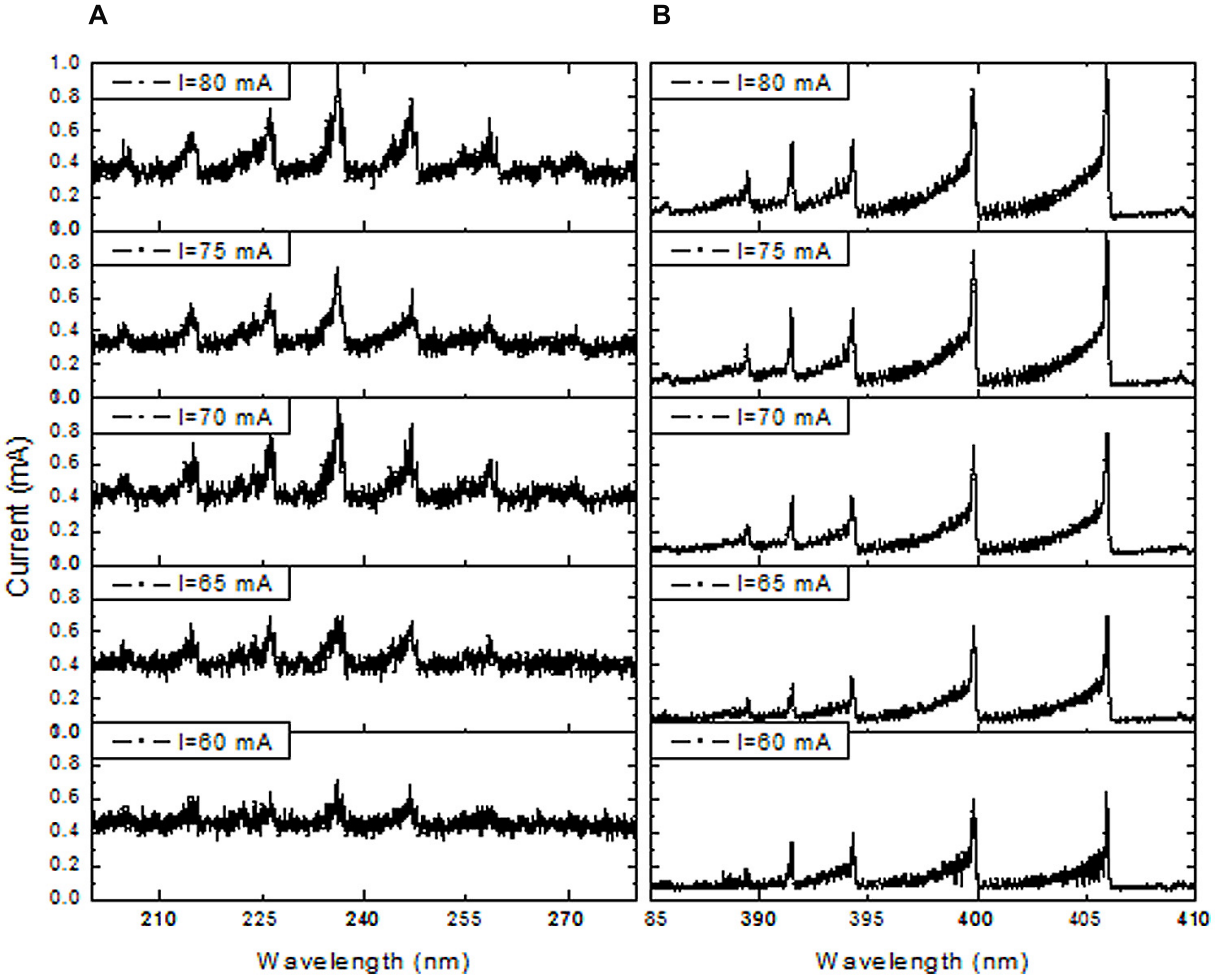
**Effect of DBD current increment on emission spectra of NO bands in the range of 200 to 280 nm **(A)** and in the N_2_ first negative system (391 nm) spectra in the range of 385 to 410 nm (B)**.

The DBD plasma emission spectra between 200 and 850 nm revealed the presence of nitrogen molecules (N_2_) (**Figure 3A**). The maximum intensity of the spectra of the nitrogen molecule second positive system (C^3^Π_u_ → B^3^Π_g_) in UV was observed at its 0–0 transition (337.2 nm). Therefore, the total spectra were normalized with respect to the nitrogen molecule (N_2_) second positive system (C^3^Π_u_ → B^3^Π_g_) 0–0 transition intensity. The emission spectra of the N_2_^+^ first negative system (B^2^ Σ_u_^+^ → X^3^Σ_g_^+^) were measured at 391 nm, indicating the presence of a high electron temperature in the generated DBD plasma. Moreover, the Nitric oxide (NO) radical emission spectra increased with higher DBD current values (**Figure 3B**). NO and N_2_^+^ production increased with increasing discharge current (**Figures 4A,B**). The existence of the N_2_^+^ first negative system and NO radical showed a high level of non-equilibrium in the generated plasma (non-thermal). The presence of high electron temperature electrons (energetic) initiates dissociation and ionization, which are essential in bio-decontamination. The increase in the emission spectra of the NO and N_2_^+^ first negative system indicates an increase in their contribution to the decontamination with increasing DBD current.

### Evaluation of Wastewater Bacterial Community by PCR-DGGE Fingerprinting

**FIGURE 5:**
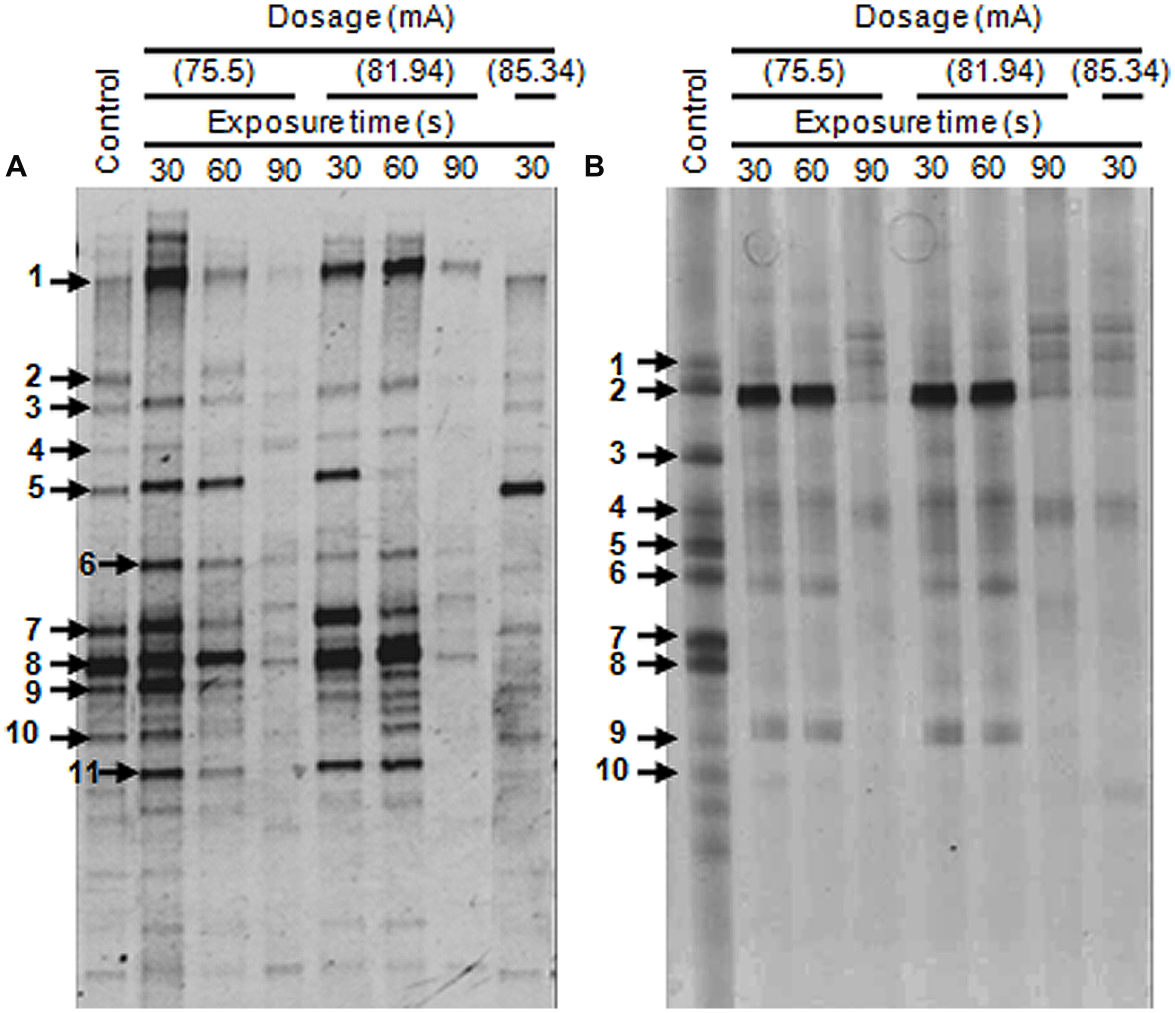
**Denaturing gradient gel electrophoresis (DGGE) profiles of 16S rDNA amplified from genomic DNA for untreated and plasma-treated wastewater samples. (A)** Samples from the food processing industry. **(B)** Samples from the leather industry. Faint DGGE bands representing polymerase chain reaction (PCR) artifacts were neglected.

Denaturing gradient gel electrophoresis profiles of the bacterial communities for the original untreated wastewaters from two different sources were determined. Eleven major DGGE bands designated as DGGE-A1-11 were detected in the food processing wastewater (**Figure 5A**). The affiliations of these OTUs were determined by comparison of their 16S rRNA gene sequences with those in the GenBank database (**Table 1**). Sequence analysis of the selected DGGE bands revealed the predominance of OTUs affiliated with Gammaproteobacteria. Identification of predominant OTUs revealed the presence of different bacterial species including *E. coli* (DGGE-A1), *Klebsiella pneumoniae* (DGGE-A2), Coliforms (DGGE-A3), *Aeromonas* sp. (DGGE-A4, A5, A7), *Vibrio* sp. (DGGE-A6), *Pseudomonas* sp. (DGGE-A8, A9, A11) and an uncultured bacterium (DGGE-A10).

**Table 1:** Identification and phylogenetic affiliation of the 16S rDNA sequences from denaturing gradient gel electrophoresis (DGGE) bands with their highest similarity matches from NCBI database.

Wastewater sample	DGGE bands	Closest match			Accession no.	Phylogenetic affiliation
		Identity	Accession no.	Similarity (%)		
A	A1	*Escherichia coli* chzl B3	KJ540213	99	LC011117	Gammaproteobacteria
	A2	*Klebsiella pneumoniae* blaNDM-1	CP009114	99	LC011118	Gammaproteobacteria
	A3	Coliform bacterium YT3-5	KF418620	99	LC011119	Environmental sample
	A4	*Aeromonas bestiarum* OTC5b	KJ726631	100	LC011120	Gammaproteobacteria
	A5	*Aeromonas* sp. BAB-3707	KM104684	99	LC011121	Gammaproteobacteria
	A6	*Vibrio* sp. S1110	FJ457366	99	LC011122	Gammaproteobacteria
	A7	*Aeromonas hydrophila* M56	KJ877664	99	LC011123	Gammaproteobacteria
	A8	*Pseudomonas putida* PM4	KJ907483	100	LC011124	Gammaproteobacteria
	A9	*Pseudomonas* sp. RJ42	KJ831073	100	LC011125	Gammaproteobacteria
	A10	Uncultured bacterium clone ncm72	KF102102	100	LC011126	Environmental sample
	A11	*Pseudomonas stutzeri* YC-YH1	KJ786450	100	LC011127	Gammaproteobacteria
B	B1	*Enterobacter aerogenes* BMW/2E	KJ995857	99	LC011128	Gammaproteobacteria
	B2	*Pseudomonas* sp. ESBL397B1	KJ831460	99	LC011129	Gammaproteobacteria
	B3	*Klebsiella* sp. SUS9K	KF991505	100	LC011130	Gammaproteobacteria
	B4	*Citrobacter freundii* C8-19	KM222631	96	LC011131	Gammaproteobacteria
	B5	*Pseudomonas stutzeri*	KJ801856	100	LC011132	Gammaproteobacteria
	B6	*Aeromonas salmonicida* w-6	KM117163	100	LC011133	Gammaproteobacteria
	B7	Uncultured bacterium clone SY1-79	KF571773	99	LC011134	Environmental sample
	B8	*Acidithiobacillus ferrooxidans* L01	KJ648626	100	LC011135	Gammaproteobacteria
	B9	*Pseudomonas putida* P1	KJ960183	100	LC011136	Gammaproteobacteria
	B10	Uncultured *Clostridium* sp. M34B-32	AB844774	100	LC011137	Gammaproteobacteria

The DGGE patterns of wastewater from the leather industry showed the presence of 10 major OTUs designated as DGGE-B1-10 (**Figure 5B**). These OTUs were identified as members of *Enterobacter* (DGGE-B1), *Pseudomonas* (DGGE-B2, B5, B9), *Klebsiella* (DGGE-B3), *Citrobacter* (DGGE-B4), *Aeromonas* (DGGE-B6), *Acidithiobacillus* (DGGE-B8), and uncultured bacteria (DGGE-B7, B10). Although the analyzed wastewater samples were found to harbor different bacterial species, they were only affiliated with Gammaproteobacteria.

### Impact of DBD Plasma on Bacterial Community Structure

Polymerase chain reaction-Denaturing gradient gel electrophoresis of the industrial wastewaters showed large variations in band number, intensity, and diversity in response to DBD plasma treatment (**Figure 5**). The DGGE profiles in treated samples changed gradually and decreased to extinction upon plasma treatment at different ranges. The dynamics of bacterial populations in response to different plasma treatment protocols are presented in **Table 2**. No changes in food processing wastewater bacterial populations were observed following exposure to low power DBD plasma at 75.5 mA for 30 s. Bacterial community structure changed slightly when exposure time was extended to 60 s. An approximately, 10% reduction in bacterial population was observed as a result of elimination of uncultured members. The rest of the bacterial sp. were resistant and remained unaffected. Treatment for 90 s resulted in 45.5% reduction in bacterial populations with elimination of *E. coli, K. pneumoniae, Aeromonas* sp., *Pseudomonas* sp., *P. stutzeri*, and the uncultured bacterium.

**Table 2:** Dynamics of wastewater’s bacterial populations in response to different dielectric barrier discharge (DBD) plasma treatment protocols.

Plasma dosage (mA)	Time (s)	Bacterial sp. in sample (A)			Bacterial sp. in sample (B)		
		Reduction (%)	Eliminated sp.	Detected sp.	Reduction (%)	Eliminated sp.	Detected sp.
control	–	0	–	All	0	–	All

75.5	30	0	–	All	50	*Enterobacter aerogenes* *Klebsiella* sp. Uncultured bacterium *Acidithiobacillus ferrooxidans* Uncultured *Clostridium* sp.	*Pseudomonas* sp. *Citrobacter freundii* *Pseudomonas stutzeri* *Aeromonas salmonicida* *Pseudomonas putida*
	
	60	10	Uncultured bacterium	*Escherichia coli* *Klebsiella pneumoniae* Coliform bacterium *Aeromonas bestiarum* *Aeromonas* sp. *Vibrio* sp. *Aeromonas hydrophila* *Pseudomonas putida* *Pseudomonas* sp. *Pseudomonas stutzeri*	50	*Enterobacter aerogenes* *Klebsiella* sp. Uncultured bacterium *Acidithiobacillus ferrooxidans* Uncultured *Clostridium* sp.	*Pseudomonas* sp. *Citrobacter freundii* *Pseudomonas stutzeri* *Aeromonas salmonicida* *Pseudomonas putida*
	
	90	45.5	*Klebsiella pneumoniae* *Aeromonas* sp. *Pseudomonas* sp. Uncultured bacterium *Pseudomonas stutzeri*	Coliform bacterium *Aeromonas bestiarum* *Vibrio* sp. *Aeromonas hydrophila* *Pseudomonas putida* *Escherichia coli*	80	*Enterobacter aerogenes* *Klebsiella* sp. *Pseudomonas stutzeri* *Aeromonas salmonicida* Uncultured bacterium *Acidithiobacillus ferrooxidans* *Pseudomonas putida* Uncultured *Clostridium* sp.	*Pseudomonas* sp. *Citrobacter freundii*

81.94	30	18.2	*Klebsiella pneumoniae* *Pseudomonas* sp.	*Escherichia coli* Coliform bacterium *Aeromonas bestiarum* *Aeromonas* sp. *Vibrio* sp. *Aeromonas hydrophila* *Pseudomonas putida* Uncultured bacterium *Pseudomonas stutzeri*		*Enterobacter aerogenes* *Klebsiella* sp. Uncultured bacterium *Acidithiobacillus ferrooxidans* Uncultured *Clostridium* sp.	*Pseudomonas* sp. *Citrobacter freundii* *Pseudomonas stutzeri* *Aeromonas salmonicida* *Pseudomonas putida*
	
	60	18.2	*Klebsiella pneumoniae* *Aeromonas* sp.	*Escherichia coli* Coliform bacterium *Aeromonas bestiarum* *Vibrio* sp. *Aeromonas hydrophila* *Pseudomonas putida* *Pseudomonas* sp. Uncultured bacterium *Pseudomonas stutzeri*		*Enterobacter aerogenes* *Klebsiella* sp. Uncultured bacterium *Acidithiobacillus ferrooxidans* Uncultured *Clostridium* sp.	*Pseudomonas* sp. *Citrobacter freundii* *Pseudomonas stutzeri* *Aeromonas salmonicida* *Pseudomonas putida*
	
	90	63.6	*Klebsiella pneumoniae* Coliform bacterium *Aeromonas bestiarum* *Aeromonas* sp. *Pseudomonas* sp. Uncultured bacterium *Pseudomonas stutzeri*	*Escherichia coli* *Vibrio* sp. *Aeromonas hydrophila* *Pseudomonas putida*		*Enterobacter aerogenes* *Klebsiella* sp. *Pseudomonas stutzeri* *Aeromonas salmonicida* Uncultured bacterium *Acidithiobacillus ferrooxidans* *Pseudomonas putida* Uncultured *Clostridium* sp.	*Pseudomonas* sp. *Citrobacter freundii*

85.34	30	45.5	*Klebsiella pneumoniae* *Pseudomonas putida* *Pseudomonas* sp. Uncultured bacterium *Pseudomonas stutzeri*	*Escherichia coli* Coliform bacterium *Aeromonas bestiarum* *Aeromonas* sp. *Vibrio* sp. *Aeromonas hydrophila*	80	*Enterobacter aerogenes* *Klebsiella* sp. *Pseudomonas stutzeri* *Aeromonas salmonicida* Uncultured bacterium *Acidithiobacillus ferrooxidans* *Pseudomonas putida* Uncultured *Clostridium* sp.	*Pseudomonas* sp. *Citrobacter freundii*
	
	60	100	All	–	100	All	–
	
	90	100	All	–	100	All	–

Treatment at 81.94 mA for either 30 or 60 s resulted in an 18.2% reduction in populations. However, elevated exposure time for 90 s resulted in a 63.6% reduction with elimination of most bacterial species. Resistant bacteria included *E. coli, Vibrio* sp., *A. hydrophila*, and *Pseudomonas putida*.

Treatment at 85.34 mA for 30 s resulted in a 45.5% reduction in populations. Bacterial populations were completely eliminated when exposure time was extended up to 60 s, as indicated by negative PCR results.

Dominant bacterial groups in leather processing wastewater changed greatly upon exposure to plasma at 75.5 mA for 30 or 60 s, with *E. aerogenes, Klebsiella* sp., *A. ferrooxidans*, and uncultured members being most sensitive. Extension of exposure time to 90 s resulted in an 80% reduction in bacterial populations and elimination of all bacterial groups except for *Pseudomonas* sp. and *C. freundii*. The same trend was observed in response to 81.94 mA treatment, suggesting that resistant strains could be selected by plasma treatment at this dosage. The bacterial community was greatly affected upon treatment with elevated plasma dosage, and all bacterial groups were eliminated upon treatment with 85.34 mA for either 60 or 90 s.

### Effects of DBD Plasma on Diversity, OTUs Richness, and Cell Viability

**FIGURE 6:**
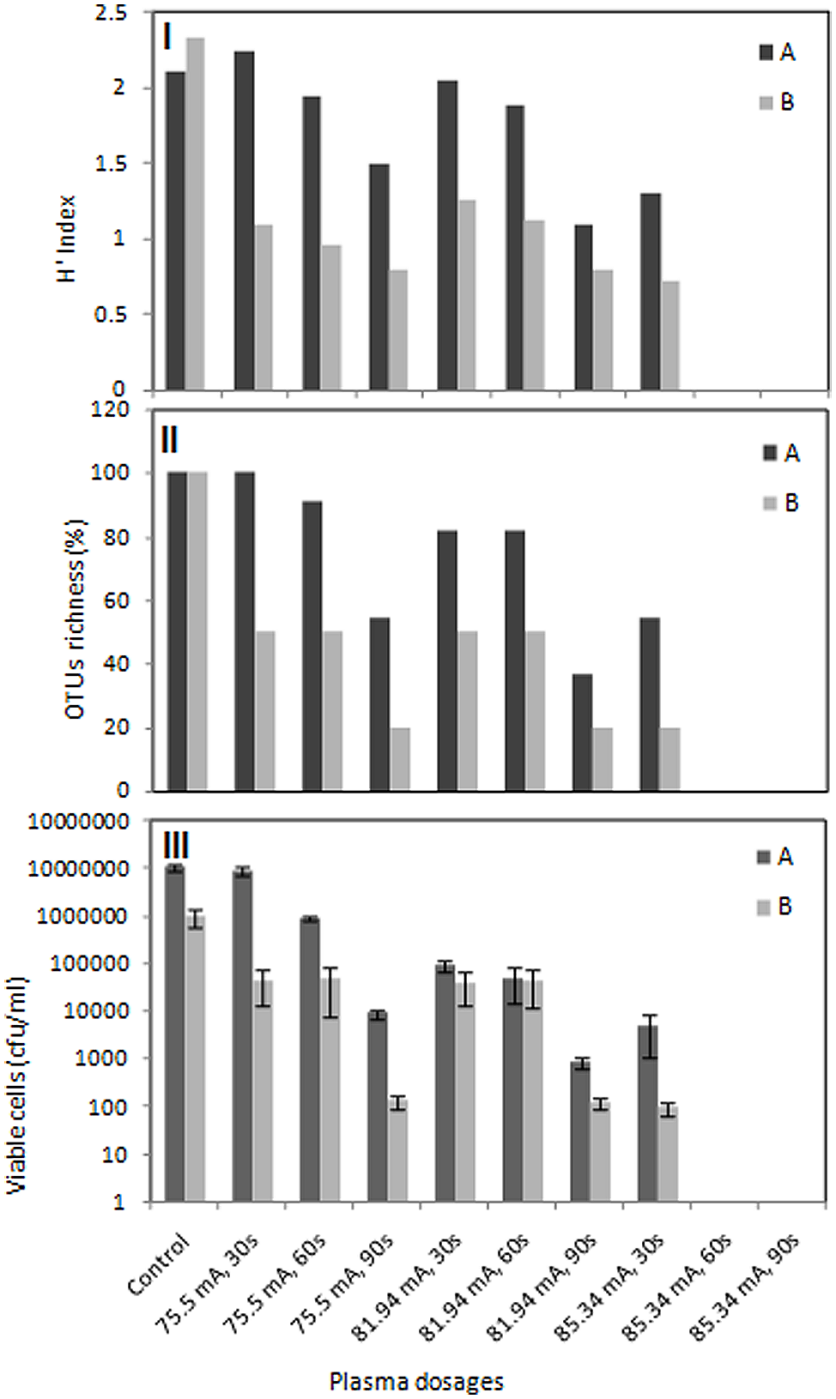
**Effects of DBD plasma on diversity, OTUs richness and cell viability.** Numerical analysis of the DGGE fingerprints to determine diversity represented by *H* index **(I)** and OTUs richness **(II)** as well as viable cell counts **(III)** of bacterial populations in food processing (A) and leather processing (B) wastewaters. Viable cell counts are represented as means of three independent enumerations (±SD).

Shannon diversity index and species richness are two parameters for estimation of bacterial diversity in environmental samples. In this study, *H* index was used as indicator for bacterial diversity (**Figure 6I**) and species richness (%) was presented as a function of relative abundance of the detected OTUs (**Figure 6II**). The bacterial community of untreated food processing and leather wastewaters showed a diversity index of 2.11 and 2.33, respectively. Diversity of wastewater bacteria from both industries was greatly affected by plasma treatment. Bacterial diversity in food processing and leather industry wastewaters was gradually decreased to 1.5 and 0.8, respectively at 75.5 mA treatment for 90 s. The same trend was observed for treatment at 81.94 or 85.34 mA. Diversity was reduced to 1.3 and 0.72 for food wastewater and leather wastewater, respectively, when treated at 85.34 mA for only 30 s.

The bacterial community of food processing wastewaters showed a gradual decrease in species richness to 90.9 and 54.5% when treated with plasma at 75.5 mA for 60 and 90 s, respectively. Species richness falls from 81.81 to 36.36% when wastewater was treated at 81.94 mA for 60 and 90 s, respectively. Treatment at 85.34 mA for only 30 s resulted in reduction of species richness to half. Species richness was greatly affected by plasma treatment for leather industry wastewater. Species richness falls to 50% and then 20% when wastewaters were treated with 75.5 mA plasma for 60 and 90 s, respectively. The same trend was observed for treatment at 81.94 mA. Only 20% survived when treatment was elevated at 85.34 mA for 30 s. Extension of exposure time resulted in extinction of all bacteria species in both wastewaters. Cell viability was also greatly affected by DBD plasma treatment. Increasing plasma dosage and exposure time resulted in a gradual decrease in viable cell counts for both wastewater samples (**Figure 6III**). DBD plasma treatment at 85.34 mA for either 60 or 90 s resulted in complete elimination of culturable bacteria in both wastewater samples.

## Discussion

Laroussi (1996) first demonstrated that glow discharge plasma generated at atmospheric pressure was a very effective sterilization agent. Since then, plasma treatment has been reported to be effective for inactivation of a variety of microorganisms; however, its effectiveness differs owing to differences in membrane structure (Lu et al., 2014). Non-thermal atmospheric plasma has been used for different biological applications including sterilization of a wide range of bacteria (Deng et al., 2007a; Ermolaeva et al., 2012; Zhang et al., 2013; Ziuzina et al., 2013, 2014) and decontamination of fungi (Leclaire et al., 2008; Ouf et al., 2015). Most studies of the effectiveness of these treatments have been based on culture-based microbiological methods. Although this would be sufficient if treatment was restricted to individual strains of cultured microorganisms, this protocol is not suitable for environmental samples because most bacteria in such samples are difficult to culture (Ward et al., 1990). Recently, culture-independent approaches have been widely applied to analyses of bacterial community structure (Amann et al., 1995; Head et al., 1998; Hugenholtz et al., 1998), and DGGE provides data enabling rapid comparison of many communities (Muyzer et al., 1997). PCR-DGGE analysis utilizing 16S rRNA genes usually yields patterns that reflect the composition of dominant microorganisms, including non-culturable members (Head et al., 1998). Accordingly, this method has been introduced as a potential genetic fingerprinting technique for investigation of microbial communities in a variety of habitats and has become the method of choice when studying bacterial communities associated with environmental perturbations or seasonal changes (Iwamoto et al., 2000; Ogino et al., 2001). In this study, we employed DGGE to evaluate the impact of cold atmospheric pressure DBD plasma on changes in bacterial community structure in wastewater from two industrial activities. The Gammaproteobacteria comprise several important groups of bacteria, including the *Enterobacteriaceae, Vibrionaceae, Pseudomonadaceae*, and *Xanthomonadaceae*. They have been detected in a variety of wastewaters (Williams et al., 2010; Broszat et al., 2014). The dominant population in the food processing wastewater consisted of a variety of Gammaproteobacteria, including *E. coli, K. pneumoniae*, Coliforms, *Aeromonas* sp., *Vibrio* sp., *Pseudomonas* sp., and uncultured bacteria. Gammaproteobacteria are also known to be dominant in chromium contaminated sites such as leather processing wastewater (Chakraborty and Bhadury, 2015). The DGGE patterns of wastewater from the leather industry were dominated by Gammaproteobacteria, including *Enterobacter, Pseudomonas, Klebsiella, Citrobacter, Aeromonas, Acidithiobacillus* along with an uncultured *Clostridium* sp.

The bacterial community gradually changed and abundance decreased to extinction upon plasma treatment. Before and after plasma treatment, Gammaproteobacteria-affiliated ribotypes dominated both wastewater bacterial communities.

The bacterial community structure in food processing wastewater remained intact when exposed to low power plasma at 75.5 mA for short periods of time. However, when exposure time was extended, the bacterial community structure significantly changed owing to differences in sensitivities of the individual species. Resistant species of bacteria included *E. coli*, Coliforms, *Aeromonas* sp., *Vibrio* sp., and *P. putida*. Treatment at 81.94 mA for 90 s resulted in elimination of one coliform bacterium and survival of *E. coli, Aeromonas* sp., *Vibrio* sp., and *P. putida*. Elevated treatment of plasma at 1.785.34 mA for either 60 or 90 s resulted in complete elimination of all bacterial groups, as indicated by negative PCR results upon analysis of samples treated with that protocol.

Members of Gammaproteobacteria were also abundant in leather processing wastewater, and were recovered in most treatments. Dominant bacterial groups in leather processing wastewater changed greatly upon exposure to plasma at 75.5 mA for 30 or 60 s, whereas *Enterobacter aerogenes, Klebsiella* sp., *Pseudomonas stutzeri*, and *Acidithiobacillus ferrooxidans* were sensitive and eliminated from the community. Extension of the exposure time to 90 s resulted in elimination of all bacterial groups except for *Pseudomonas* sp. ESBL397B1 and *C. freundii*. The same trend was observed following treatment at 81.94 mA, suggesting the selection of resistant strains by plasma treatment at this level. The bacterial community was greatly affected upon treatment with elevated plasma dosages. All bacterial groups were sensitive and completely extinct upon treatment at 85.34 mA for either 60 or 90 s. Overall, low power plasma for short exposure times resulted in changes in bacterial species, while plasma treatment at high power for relatively longer times resulted in complete sterilization and extinction of all bacterial groups within the community.

The roles of various plasma agents in the inactivation of bacteria have recently been investigated (Laroussi and Leipold, 2004; Lu et al., 2008; Yasuda et al., 2010; Hao et al., 2014). Sterilization induced by DBD air plasma is believed to be due to the production of certain reactive charged particles. NO is known to be a dominant long-lived gaseous species that is generated by plasma jets. The results of the present study suggested that reactive NO radicals were generated in response to unstable DBD plasma operation as indicated by the inhomogeneity and presence of brighter channels between electrodes. In this experiment, the consumed energy increased linearly with discharge current; therefore, NO and N_2_^+^ production increased with increasing discharge current (**Figures 4A,B**). Moreover, increases in rotational temperature with discharge current are known to stimulate production of NO radicals. These results are in agreement with previously reported data for an air micro-hollow discharge plasma jet experiment in which the NO increased with increasing power (Hao et al., 2014). In this study, NO emission spectra intensity increased with discharge current, as did the effectiveness of elimination of bacterial species. NO can easily permeate cell membranes and become involved in a variety of chemical reactions that ultimately affect microbial cells. Several studies have investigated the lethal mechanisms of NO on different bacteria (Ascenzi et al., 2003; Ulett and Potter, 2011; Privett et al., 2012; Schairer et al., 2012). NO can affect many biological processes directly by reacting with proteins and other macromolecules or indirectly by forming intermediate reaction products that will eventually interfere with biochemical processes (Patel et al., 1999; Ascenzi et al., 2003). Inside the cell, NO can interact with other radicals, resulting in oxidative species such as peroxinitrite, which is known to affect cell functions in many ways (Radi, 2013) and to be a very effective bactericidal agent. NO is also able to react with metal complexes, which are important for the metabolism of bacteria (Ford, 2010).

In this study, DBD air plasma emission spectra showed the presence of N_2_^+^ particles (**Figure 3A**). An increase in N_2_^+^ production with discharge current was observed in this study (**Figure 4B**), which was attributed to increasing current density and electron density. The increase in electron density led to an increasing ionization rate, resulting in an ultimate increase in the production rate of N_2_^+^ (**Figure 4B**). Deterioration of the wastewater bacterial community structure was found to be consistent with increases in N_2_^+^ production, especially when the treated sample was placed on the ground electrode that accelerated the charged N_2_^+^ particles toward it. Bacterial inactivation in response to treatment with a He plasma jet containing N_2_^+^ particles was previously reported (Seo et al., 2010). Moreover, charged particles such as N_2_^+^, O_2_^+^, and O_2_^-^ have been shown to play a very significant role in rupture of the bacterial cell outer membrane owing to the electrostatic force caused by charge accumulation on the outer surface of the cell membrane exceeding the membrane tensile strength (Tiwari et al., 2009).

UV radiation in the range of 200–300 nm with several mW-s/cm^2^ doses is known to cause lethal damage to cells due to dimerization of thymine bases in their DNA strands, which inhibits the ability of the cell to replicate properly. However, UV emitted from N_2_ and NO bands in this study was weak with respect to the emission from 300 to 400 nm (**Figure 3A**). Overall, these findings indicate that UV played no significant role in the bacterial inactivation process. Moreover, generated APP UV radiation was reabsorbed in the plasma volume; therefore, the cell death due to plasma exposure cannot be attributed to UV radiation (Philip et al., 2002; Laroussi and Leipold, 2004; Gaunt et al., 2006).

## Conclusion

In this study, the impact and role of DBD plasma on bacterial community structure and elimination of wastewater bacteria was investigated. The results revealed large variations in microbial diversity in response to different plasma treatment protocols. Specifically, the wastewater bacterial community gradually changed and decreased to extinction in response to increasing plasma dosage over extended exposure time. No significant changes in food wastewater community structure were observed in response to short-term plasma irradiation at lower dosages; however, elevated plasma dosage for even a relatively short time destroyed most of the bacterial populations, and extension of exposure time resulted in elimination of all bacterial groups. Dominant bacterial groups in leather processing wastewater were much more sensitive to plasma irradiation, with most bacterial populations being affected at lower plasma dosages and completely eliminated at higher dosages. The effects of DBD plasma on bacterial populations were attributed to the generated charged particles. In addition to being detected, both NO and N_2_^+^ production rate increased with increasing discharge current. NO and N_2_^+^ are known for their crucial role in the inactivation of the bacteria; however, the results of the present study indicated that they played little or no role in the sterilization process. Taken together, the results of this investigation indicate that atmosphere pressure DBD plasma is a promising tool for wastewater treatment owing to its ability to eliminate almost all of the enclosed bacterial populations within a short period of time.

## Conflict of Interest Statement

The authors declare that the research was conducted in the absence of any commercial or financial relationships that could be construed as a potential conflict of interest.

## Supplementary Material

Figure S1 **Measurement of applied voltage, total current, and ground current waveforms of atmospheric pressure DBD plasma in air**.
