# Supplementary figures and images for: Deterioration to extinction of wastewater bacteria by non-thermal atmospheric pressure air plasma as assessed by 16S rDNA-DGGE fingerprinting
Source: Front Microbiol. 2015 Oct 6;6:1098. doi: 10.3389/fmicb.2015.01098 (PMC4594161; doi:10.3389/fmicb.2015.01098)

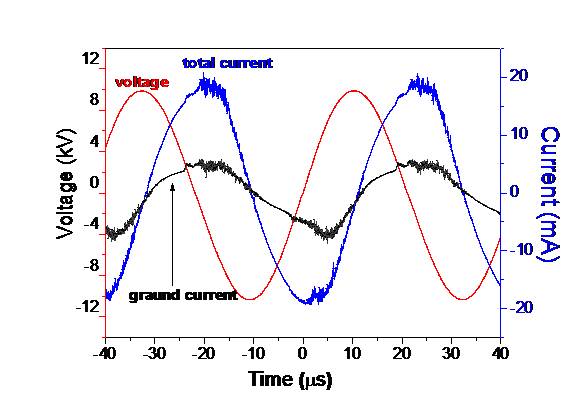

Supplement: Figure S1 — Measurement of applied voltage, total current, and ground current waveforms of atmospheric pressure DBD plasma in air. [file Image_1.JPEG]
